# The tensions between micro-, meso- and macro-levels: physiotherapists’ views of their role towards fall prevention in the community – a qualitative study

**DOI:** 10.1186/s12913-020-4940-1

**Published:** 2020-02-06

**Authors:** Sara Cerderbom, Maria Bjerk, Astrid Bergland

**Affiliations:** Faculty of Health Sciences, OsloMet - Oslo Metropolitan University, 0130 Oslo, Norway

**Keywords:** Fall prevention, Communication, Empowerment, Implementation, Older people

## Abstract

**Background:**

Falls are a global public health concern. Physiotherapists are a key resource in this context, but there is sparse knowledge about how they perceive their role in the primary care setting. Therefore, the purpose of the present study is to explore physical therapists’ (PTs) view of how they experience and perceive their role working with fall prevention in a community care setting.

**Methods:**

Semi-structured interviews were conducted with 17 physiotherapists. Data were analysed using a qualitative thematic analysis.

**Results:**

The analysis resulted in a core theme and three subthemes. The core theme was ‘capability to cope with the tensions between the micro-, meso- and macro-levels in fall, prevention’, which indicated the importance of an evolving multifaceted, evidence based and innovative physiotherapy role. A key factor for this role is to take an integrative biopsychosocial approach based on how biological and psychosocial factors are uniquely related in fall prevention. The three themes were as follows: 1) always moving and changing: the competent explorative knowledge-hungry clinician’s multifaceted role; 2) multiprofessional – but in the end alone; 3) reaching out *–* from the bottom to the top. Success in the role of physiotherapists in fall prevention depends on the empowering leadership and working culture, as well as on the time and multifaceted professional competence of the clinicians.

**Conclusion:**

Our findings indicate that the PTs’ role reflects their abilities to change and improve their professional work in accordance with evidence based knowledge. To ensure good quality the PTs focused on the special needs of the patients, evidence-based fall prevention, interdisciplinary team work, good clinical competences, good skills in communication, and interpersonal relations. Attention should be placed on the importance of biopsychosocial perspective framing in the actual clinical and political context. The PTs saw the need for working at the micro-, meso- and macro-levels to succeed in the work of fall prevention.

## Background

Falls pose a major threat to the well-being and quality of life of older people and have become one of the most important global public health concerns for community-dwelling older people and society in general [1]. Every year, 30% of community-dwelling older people experience a fall, and falls are a leading cause of morbidity, mortality, functional disability, hospitalisation and institutionalisation in older adults [2, 3]. There is robust evidence that exercise can reduce falls in the older population [4]. Several systematic reviews and meta-analyses have stated that exercise is one of the most powerful ways to prevent falls and that physiotherapists (PTs) play a multifaceted role in this context [4–6]. Because falls are multifactorial, fall prevention also must address biopsychosocial characteristics such as age, history of previous falls, health conditions, polypharmacy, pain, fear of falling, self-efficacy for exercise and social support [2, 7–9].

However, fall prevention is an important topic for a variety of professional groups working with PTs, such as PTs assistants occupational therapists, nurses and physicians [2, 10]. In many Western countries; therefore, politicians engage with this topic. Previous research has shown that the most powerful means of preventing falls is exercise [5], and PTs often provide this type of intervention, playing a significant role in fall prevention [11].

To foster higher levels of consciousness about fall prevention among those at risk, more qualitative research can provide a deeper understanding of the development and experience of fall prevention work. Hence, the present study aimed to explore PTs’ view of how they experience and perceive their role in preventing falls in the primary care setting.

## Methods

### Design

The current study used a qualitative design with a phenomenological perspective. The goal of a phenomenological perspective is to summarise individual experiences and provide descriptions that include ‘what’ people experience and ‘how’ they experience it [12]. The consolidated criteria for reporting qualitative research (COREQ) were used to guide the reporting of the current study [13].

### Informants and setting

To answer the research question, we used a purposive sample, which are used when the opinion of experts in a particular field is the topic of interest [14]. The current study was conducted in six municipalities in Eastern Norway, where the participating PTs had earlier been involved in a randomised controlled trial evaluating a fall preventative exercise programme [15, 16]. All informants had clinical experience in the area of fall prevention and working with older adults. Table 3 presents information about the participants’ age, gender, years of experience as PTs, years of experience in the primary healthcare service, additional education and position and/or unit. In addition, to participate in the present study, the informants were required to speak and understand Norwegian.

The second author contacted the informants by telephone and gave oral information about the study and invited them to participate. If consent to participate was given, the interview was scheduled by the last author.

### Ethical considerations

The study was conducted in accordance with the principles of the Declaration of Helsinki of the World Medical Association [17]. Verbal information about the study was provided for the informants, and oral consent was gathered from all the informants before to data collection began. Written consent was also obtained prior to data collection. The informants were guaranteed confidentiality and reassured that their participation was voluntary and that they could withdraw from the study at any time without needing to state their reasons for doing so. The study was approved by the Regional Committee for Medical Research Ethics in South Norway (Ref. 2014/2051).

### Data collection

One team member (AB), experienced in qualitative methods, conducted all semi-structured interviews. The interviews were based on an interview guide created by the authors, see Table 1. Before beginning the interview, the interviewer told the informant about the purpose of the interview. All the interviews were introduced in the same way, starting with the following question: ‘Overall, what role do you play as a PT while working in fall prevention in the municipalities?’ To help the informants talk freely about their experiences of working on fall prevention in a primary care setting, the interviews were conducted in a dialogue form and comprised of follow-up questions based on the participants’ answers to the main questions [18]. Their responses were recorded and used in the follow-up questions. We aimed to recruit between 10 and 20 patients or to continue recruitment until we reached data saturation [14, 19] to ensure what Malterud et al. [20] describe as information power. The goal was to obtain cases deemed rich in information for the purpose of saturating the data [21]. The interviews were conducted over a period of 5 months (October 2016 – February 2017), and each interview lasted for about 1 h and was conducted at the interviewer’s office.

**Table 1:** Examples of questions in the interview guide

Please, describe what overall role do you play as a PT while working in fall prevention in the municipalities? Please, describe your thoughts on the importance of theory and evidence based practice in fall prevention Please, describe your thoughts on the role of context in fall prevention Please, describe your thought on the role of organization in fall prevention Please, describe your thought on multiprofessional work in fall prevention	

### Data analysis

The interviews were audio recorded and then transcribed verbatim. The transcriptions from the 17 interviews totalled 253 pages. The data were analysed using the content thematic analysis by Braun and Clark [22]. We can never be entirely be free of preconceptions that could influence our interpretation of the data. Therefore, to strengthen the validity, we addressed the criteria of trustworthiness, which include credibility, dependability, confirmability, transferability and authenticity [23]. To fulfil this, all the authors, physiotherapists with years of experiences in fall prevention research, made their preconceptions and existing knowledge about the context explicit and carried out the analysis based on the following six stages [22]:

1. Become familiar with the data. All four authors separately read and reread the transcripts and noted codes. In a face-to-face meeting, all the authors discussed the overall understanding of the data to share their comprehension and compare their notes for essential meanings.
2. Generate initial codes. All the authors noted initial codes on the transcripts manually and separately. They then met to compare codes and construct a mutual coding tree.
3. Search for themes. We identified central quotations that we inserted into a common matrix, with the following headlines: quote, our understanding, theme and subtheme/candidate theme (Table 2).
4. Review themes. The research group met to compare and discuss the themes. We used yellow stickers to highlight themes emerging from each focus group and used a blackboard to summarise the findings. Thereafter, the authors compared the findings across all groups. One important step was to explore the similarities and differences between the group’s answers on the same topic.
5. Define and name themes. This stage was a back-and-forth process involving mutual reflections by the researchers involved in coding and further discussions of the findings with all authors, resulting in the final form reported in the present paper.
6. Produce a report with all the other authors involved. Example of the analysis procedure is presented in Table 2.

**Table 2:** Example of the analysis procedure from meaning unit to theme

Meaning unit	Codes	Theme
It’s about supporting a behavioural change … that is, a change in which the patient somehow takes his behaviour and its consequences into consideration. They must make up their mind about it, make decisions about how they want to live their lives. Understanding is so important. We must respect the person’s autonomy while exploring the opportunities for behavioural change. It may also be conceivable that a lack of adherence with what we recommend is because the person does not realise that it is a problem. We need to think about underlying psychological factors in change and help even more people manage to implement our interventions. It is important to use evidence-based knowledge or best practice	Behaviour change	Always moving and changing: The competent, explorative and knowledge-hungry clinician’s multifaceted role
	Active listening	
	Communication	
	Respect autonomy	
	Support behaviour change	
	Empty	
	Motivate	
	Adherence	
	Use evidence-based knowledge	
	User’s understanding	
	Psychologic knowledge	
	Knowledge translation	
	Best practice	

## Results

The citations in the results are provided with numbers, which refer to the number of the PT in Table 3.

**Table 3:** Characteristics of the Study Participants’

Physio- therapist	Age groups in years	Gender	Years of experience	Years working in primary healthcare	Additional education	Position/Unit
1	30–34	Male	2	2	Sports teacher	Rehabilitation
2	30–34	Male	6	6	Physiotherapy for older adults	Rehabilitation
3	30–34	Female	3	1.5	None	Rehabilitation
4	35–39	Female	13	7	None	Rehabilitation
5	20–24	Male	1	0.5	None	Rehabilitation
6	40–44	Male	8	2	Health communication	Rehabilitation
7	45–49	Female	19	15	Health communication	Manager physiotherapy unit
8	35–39	Female	12	12	None	Rehabilitation and preventative care
9	30–34	Male	5	5	Physiotherapy for older adults	Rehabilitation and preventative care
10	40–44	Female	16	14	Physiotherapy for older adults	Rehabilitation and preventative care
11	35–39	Male	15	15	Physiotherapy for older adults	Rehabilitation and preventative care
12	45–49	Female	25	14.5	Health communication	Reablement
13	35–39	Female	12	10	None	Rehabilitation
14	50–59	Female	25	25	Physiotherapy for older adults	Rehabilitation
15	60–69	Female	40	4	Mental health work	Rehabilitation
16	35–39	Female	10	4	None	Reablement
17	25–29	Female	4	3	None	Rehabilitation

The analysis in the current study resulted in one core theme and the following three themes:

1. Always moving and changing: The competent, explorative and knowledge-hungry clinician’s multifaceted role
2. Multiprofessional – but in the end alone
3. Reaching out *–* from the bottom to the top

The core theme revealed was ‘Capability to cope with the tensions between the micro-, meso- and macro-levels in fall prevention’. This core theme indicated the importance of an evolving multifaceted, evidence based and innovative physiotherapy role. This multifaceted and innovative role requires competent experienced and skillful physiotherapists who can cope with the interaction between the micro- (individual), meso- (organisational) and macro-levels (policy), showing that physiotherapists employed in fall prevention require a biopsychosocial approach. The analysis disclosed that PTs saw themselves as playing a crucial role in the prevention of falls in older people. They described how their multifaceted, evidence-based and innovative role means initiating and motivating both on the individual level (with the older person), with their team consisting of colleagues and at the level of the organisation (policy, work culture). Last but not least, success in the role of PTs in fall prevention depend on empowering leadership and a working culture, along with time and multifaceted professional competence. The findings below are presented according to the themes and illustrated with quotations from the interviews.

### Always moving and changing: the competent, explorative and knowledge-hungry clinician’s multifaceted role

Overall, the PTs saw themselves as having a central role in the context fall prevention because of their specific knowledge about potentially useful exercise interventions and evaluations, as well as knowledge of the biopsychosocial factors influencing patient decisions and motivation for participating in interventions. One PT expressed it as follows:

*I think it's great. I feel I have a lot of knowledge that I convey and can and should convey to those I meet (the older persons) about fall prevention. Both experience and in theory which represent our foundation for the physiotherapists’ analysis, judgement, reflection and synthesis. In our education there is a great focus on critical thinking. I can say a lot about exercise, testing, motivation, ageing and evaluation. I try to be in accordance with the current* evidence-based practice. *Some clinicians lack expertise and have limited understanding of scientific research. However, I think most PTs’ are very concerned about improving their level of scientific understanding and want to perform an evidence –based practice. (PT15)*

The PTs had learned that their role required many different competences and skills; therefore, their role was seen as multifaceted and complex. The competence and skills included more than basic competence and knowledge about the body, how to strengthen the older person’s physical performance and balance or to adapt and individualise exercises of importance for social participation. One PT said the following:

*I can talk a lot about balance, in relation to different types of balance – difference in requirements that are placed on balance when standing – by moving for different activities, such as to standing up from a chair or while walking, or when stretching for something – additionally the connection between cognitive functions and the importance of balance, attention, etc. Many of our patients are restricted in their social participation because of poor balance and being afraid of falling. The ability to stand, to walk and to go about daily activities in a safe manner depends on a complex interaction of for example physiological mechanisms, then many systems need to be evaluated to understand what is wrong with a person’s balance as well as cognition and the context. Yes, there is a separate academic landscape regarding different perspective on balance . (PT7)*

The informants also perceived that they had to have knowledge and skills about psychological and social aspects, such as motivation and support for behavioural changes, for example, to decrease fear of falling and improve self-efficacy. Moreover, to succeed in their intervention, the PTs highlighted the importance of being active listeners and communicators, building trustworthiness and a good relationship with the older person. In addition, they noted that they should also be able to translate their knowledge and competence into understandable and operational user knowledge. The following extract describes how one PT worked with behavioural changes, trying to motivate the older adult while respecting their autonomy:

*It’s about supporting a behavioural change … that is, a change in which the patient somehow takes his behaviour and its consequences into consideration. They must make up their mind about it, make decisions about how they want to live their lives. Understanding is so important. We must respect the person’s autonomy while exploring the opportunities for behavioural change. It may also be conceivable that a lack of adherence with what we recommend is because the person does not realise that it is a problem. We need to think about underlying psychological factors in change and help even more people manage to implement our interventions. It is important to use evidence-based knowledge or best practice. (PT3)*

Another PT described how she had to be sensitive to the older person’s needs and will while having an awareness of the importance of active listening, as well as building trustworthiness and a relationship between her and the client:

*There are many who do not have that background in exercising, and then a little more motivation and information is required. It’s interesting – creating a good trusting alliance between me and the patients is important. I have to be sensitive and observant. I have to listen for hints and concerns. I must also try to pick up uncertainty – it’s the patient’s own decision that counts. (PT4)*

Furthermore, the importance of external factors was mentioned. One therapist said the following:

*After all, the individual’s risk can be linked to physical and psychosocial conditions. In assessing individual risk, it is of course important how vulnerable the person is, and how many factors and risks there are in the environment he or she faces – family relationships, etc. (PT8)*

One task that helps to prevent falls is fall registration, and healthcare providers in the community are obliged to do this. Fall registration is also often the way that PTs get information about someone who is in need of an intervention. Clearly, this task was seen to be highly important and not only at the individual level. However, if fall registration is to have any effects, there must be a systematic plan of what to do with this information; otherwise, the registration is worthless, as one PT said:

*It is very important, and I wish all politicians and those who are responsible for the economy could see that there is a real problem in the community, and not only in the nursing homes and suchlike. I do not know how much notice is taken of it, but I have not seen them do more, no, I have not. Falls should be registered in a systematic way – based on evidence based practice ..but if they are registered, there should be some follow-up interventions. If the registration has no consequences, then it is wasted and demotivating. (PT11)*

### Multiprofessional – but in the end alone

To succeed with fall prevention, the work requires a multiprofessional and multidisciplinary approach, which the informants also confirmed. However, they sometimes experienced that other team members lacked evidence-based knowledge and sufficient competences to critical reflection. Furthermore the physiotherapists stated indications on that the multidisciplinary team often did not understand or had the possibilities to realize the importance of taking responsibility for the task and did not recognise or ignored that an older person was in need of fall prevention. One PT expressed the following:

*As I see it, I wish that the formal caregivers, for example, were more involved in the task and took more responsibility as a team to see that they can make a difference when they visit the care recipients so many times per day. Many of them have already changed how they work. Since the risk of falling is multifactorial the teamwork is very important and the team had to be concerned about what represent evidence based practice. They should focus on the user’s resources and not always just go in and lift them off the bed. But instead, they should be getting them to do it by themselves. But at the same time, many of them are good at their job and see the interdisciplinary part as important. They might see things that we don’t see, probably in daily tasks. So that they (the staff) come up with ideas and tips for something that they (the older persons) have problems with or could be trained to do, or tell us about it, or that they (older people) could take the initiative themselves. That is how it is! In addition I miss the possibilities for the team to sit together and discuss critically how to practice fall prevention and how to utilise one’s knowledge and critical thinking skills which is the hallmarks of clinical reasoning in physiotherapy. Actually, all healthcare providers involved in care work can operate in an interdisciplinary way around fall prevention. (PT16)*

The experience of other team members lacking knowledge also resulted in a perception that the PTs’ work was not prioritised to a high degree by other healthcare professionals. This led to a feeling of standing alone with their responsibility, as one PT described:

*My experiences from several places is that exercise is not being followed up. There is a note in the diaries or on lists that this should be followed up. and that this is an exercise and it is important for it to be followed up, but it does not seem that people understand that it is important, and then it is not always being followed up. Good role models are important. I think physiotherapists who care for older people now constitute a skilled group, which has a good reputation – that’s fine. They are interested in quality improvement. If the attitude had been that exercise was as important as medication, then it would have been followed up. Exercise is used far too little, even though it has well-documented results based on review and metanalysis. (PT5)*

A group that the PTs really noticed is missing from the team was general practitioners (GPs), and they thought that GPs lacked competence, not only in fall prevention, but also in knowledge about how to work in a team and see the importance of teamwork. One PT described this as follows:

*… Many also lack knowledge and seem unable to work together with other professionals, I think. They are just in their box, and we have tried several times to get in contact with the relevant GP through the municipal GP. We have even pushed a little. Could we not participate in a weekly meeting with the municipal GP and the relevant GPs and talk about this for perhaps ten minutes somehow? But we are met with closed doors, and as the municipal GP says, there is nothing in it for them, and the GPs who would come to these meetings are the GPs who already know about this and know that the municipality has preventative interventions to offer… .* (PT17)

Finally, in favour of succeeding with the implementation of fall prevention, the team’s views on knowledge and research were seen as playing an important role:

*In order to implement it, it must be entrenched among the leaders and in the culture of which the health profession is part. Implementation of research-based knowledge depends on the views the interdisciplinary team has on research and knowledge on research and the quality of research. (PT10)*

### Reaching out *–* from the bottom to the top

Looking at the societal level, the last theme describes how the PTs perceived the importance of reaching out with their knowledge to clinicians, leaders and policy makers. A key area that all PTs talked about in this context was the essential task of building their practice and competence on evidence-based knowledge, regardless of whom the practice or competence should be presented/transferred to. However, how much time the PTs had to update themselves depended on, for example, the leadership, working culture and how much time they had to update themselves. One PT noted the following:

*I think that one should focus on it to a greater extent in the municipalities. After all, I can see that in hospitals, for example, there is a larger academic environment, and because it involves doctors and others who may be more concerned with recent research, that then one automatically becomes part of an environment where there is a lot of professional activity. In the municipalities, it is easier to just continue with what has always been the case. I think innovation based on research is important. I think physiotherapists are interested in innovative solutions understood as something that is both useful and new as well as* evidence-based practice*. It is a little up to each department: how much time you spend on, for example, having meetings, and on up-dating yourself, simply. (PT2)*

Implementing fall prevention and reaching out to policy makers was not seen as an easy task when there was perceived to be a huge gap between themselves and policy makers. In addition, the PTs perceived there was little time to fit this in the working day:

*Implementation has to do with capacity. After all, we experience barriers attached to various important stakeholders such as managers and health professionals regarding understanding and insight into research. But our resources are a serious problem. We are too few! (PT4)*

Another stated the following:

*I feel we are appreciated and listened to, but the framework is too narrow – the economy is too poor. (PT1)*

Finally, the informants also experienced the need to be on the frontline and think more like an entrepreneur. They also perceived how in the end, their role in fall prevention could contribute to preventing the institutionalisation of older people. A PT mentioned the following:

*And then I think that professional development has an important role. Thinking that we are doing work that is going to be future-oriented means that you always have to keep up and re-orient yourself. You have to be a little quick and on your toes. Note that the fall prevention interventions are cost-effective and help older people to stay at home longer. I learned once that it pays to come up with bar graphs, because you see more than words. Yes, it has become very economical now. It is all about economics. We have to get them home as soon as possible, to prevent institutionalisations. (PT13)*

## Discussion

To the best of our knowledge, this is the first study to explore PTs’ views of their role in fall prevention in a community care setting. The overall results highlight that the PTs’ role must reflect their abilities to change and improve their professional work in accordance with evidence based knowledge. The PTs focused on the special needs of the patients, evidence-based fall prevention, good clinical competences, good skills in communication, and interpersonal relations. They stated the importance of biopsychosocial perspective [24] framing in the actual clinical and political context while calling for improved evidence-based competencies within the interdisciplinary team and increased motivation for teamwork. The importance of promoting critically informed thinking, encouraging ideas from diverse disciplines and providing a space for ideas that promote a more positive future for the profession were highlighted. The findings also show how the PTs saw the need for having communication, and social and personal competencies as well as working at the micro-, meso- and macro-levels to succeed in the work of fall prevention [25].

The findings from the current study can theoretically be connected to the biopsychosocial perspective [24] because the PTs talk about the importance of *respect for the person’s autonomy, while exploring the opportunities for behavioural change as well as the importance of the therapists thinking about underlying psychosocial factors.* From the biopsychosocial perspective, the PTs’ role presents itself in the context of a complex and multifaceted interplay of a patient’s physiological, emotional, cognitive, behavioural and sociocultural factors. These factors are important in the interaction with patients.

Our informants experienced *themselves as a skilled group with a good reputation and interest in quality improvement. They were concerned about working systematically and that their interventions would be followed up, and they used words such as ‘*future oriented’, keeping their knowledge updated and reaching out with their knowledge at a societal level. Indeed, professional competence is often described as the ability to use knowledge, skills and attitudes in a certain professional context. In ‘good’ or ‘expert’ practice, several factors are considered important, including, in line with Slade et al. [25], a person-centred approach, advanced clinical reasoning and analytical capability and patient education. The participants in the current study were pointing to the importance that professional competence is regarded as complex and, according to Bowden and Marton [26], viewed as a number of separate competences specifically needed in a professional area, here being fall prevention.

The PTs revealed, in line with Slade et al. [25], that each separate competence might consist of theoretical knowledge, practical skills and reflective abilities, which can be displayed in the acts of the professionals in a specific setting.

Our findings fit with the systematic review by Muddle et al. [27], suggesting that the evolving role of a primary healthcare practitioner demands more than just strength in the biological sciences and clinical skills: clinicians must also be able to communicate effectively. The PTs saw the importance of being active listeners, building trustworthiness and good relationships. Better treatment outcomes and experiences for both the patient and health professional were seen as relying on effective clinical communication skills for a successful interaction. Our findings illustrate the importance of *creating good alliances* and a mutual understanding of communication at the micro-, meso- and macro-levels and between these levels as well.

A positive policy environment at the macro-level is required to support the meso- and micro-levels of fall prevention; this includes leadership, financial support and human resources allocation. At the micro-level, in line with our findings, Finset [28] argues that an important aspect of person-centred communication is active listening to the patient narrative, which includes sensitivity to patient cues and concerns. Accessible, attentive care and the physical and psychosocial environment are important aspects in establishing an empowering atmosphere, which is understood as a recognition of the patient’s needs [29, 30]. Opsommer and Schoeb [31] stated that the importance of communication arises from assumptions about how information should be transmitted, how questions are designed and how treatment interventions are recommended to patients. Our results also correspond well with the ideas of Opsommer and Schoeb [31], who state that collaboration, participation, negotiation and taking the patient’s perspective into account are salient aspects of effective communication. Indeed, one PT claimed that it is the ‘*patient’s own decision that counts’.* However, the PTs were concerned about how to motivate and support behavioural changes. In healthcare systems, quality care has become synonymous with effective communication [32]. The definition of effective communication includes attending, listening and being present to show respect for the values, needs and preferences of another person [33]. Our informants talked about the importance of *respect for the person’s autonomy while exploring the opportunities for behavioural change.* For patients, a ‘good’ PT is one who is competent in interpersonal skills, manners and teaching ability and who can display effective communication skills [34, 35].

The informants were very concerned about evidence-based knowledge and *systematic reviews.* Here, evidence-based practice (EBP) refers to the translation and integration of the best available research-based knowledge, clinical expertise and patient characteristics and preferences [36]. In this manner, people can receive the most effective evidence-based healthcare [37–39]. The informants stated that a barrier towards putting research-based knowledge into practice was that they needed more resources. Our findings support the proposal of Snöljung and Gustafsson [40], who note that the barriers encountered by PTs in their implementation of EBP may be related to a lack of expertise and a limited understanding of scientific research. Further, in accordance with our findings, previous studies have also shown that most PTs are interested in improving their EBP skills and that they have a positive attitude towards EBP [41, 42].

The PTs talked about team members having a lack of evidence-based knowledge. Our informants stated the increased need for evidence-based knowledge among all team members, which is in line with the global emphasis on the need for applying research-based knowledge in clinical practice [37–39]. Furthermore, Van Rhyn and Barwick [39] conclude that practice should be evidence-based to reduce the frequency of falls, which fits it with the message of our informants, who were concerned about *professional development and working* with *evidence-based* methods*.* Hunt [43] argues that EBP is also important when professionals work as a team because it helps advance research to establish better methods of patient care. Overall, Perraton et al. [44] note that assisting with the PTs’ application of research-based knowledge in clinical practice can help enhance the quality of clinical practice and encourage lifelong learning and professional progress.

Based on our study, leadership was viewed by some as the solution for the challenges of succeeding with EBP. In line with our informants, McKeever and Brown [45] conclude that effective leaders are those who are supportive of their teams and take an active role in the work or practice changes. As stated by one of our participants, the context is a critical element in the successful implementation of evidence into practice [46]. The most frequently cited and impactful organisational barriers include a lack of administrative and cultural support (e.g., resistance from colleagues, leaders or other professionals) and insufficient resources [47, 48], which were by the participants in the current study. Indeed, overall, organisational support can facilitate EBP [49]. In the context of EBP, we would also like to highlight how our participants seemed to identify the importance of critical thinking and a comprehensive knowledge based on their work with *fall prevention. They talk about the importance of experience and theory regarding exercise, testing, motivation, ageing, evaluation, different types of balance – the differences in the requirements that are placed on balance when standing – by moving under different activities and the connection between cognitive functions.* In this context, critical thinking is the ability of a clinician to analyse complex data and situations to implement the appropriate strategies or actions, and it is necessary for effective problem solving and decision making in the clinical arena [50]; this requires health professionals to recognise relevant information, something that was emphasised by our informants as critical thinking. Terms such as clinical decision making, critical judgement and diagnostic judgement are surrogate terms often used interchangeably with each other and the term critical thinking [51]. A comprehensive knowledge base is a prerequisite for critical thinking and forms the foundation on which the cognitive process is built [51].

Another important part of our findings is that the participants indicated a positive attitude towards interprofessional competences. The World Health Organisation [52] emphasises the importance of future healthcare workers’ interprofessional competencies in optimising the skills of diverse team members, enhancing cooperation in case management and providing better healthcare for patients [53]. Interprofessional collaboration has been considered beneficial in health services for patients with chronic diseases [54].

In line with Barker, the PTs in the current study emphasised the impact of multidisciplinary, transdisciplinary working and interagency working in developing and delivering high-quality, patient-centred care [55]. ‘Multidisciplinary team working is essential in order to provide high-quality patient care’ [56] as stated in our finding with the argumentation that *the risk of falling is multifactorial* thus *the teamwork is very important*. However, the PTs seemed to have trouble with mobilising the teams, and medical doctors were mentioned as a particular issue. The PTs wanted stronger involvement from GPs because GPs have a key role in the medical care of older individuals. The importance of teamwork is important for achievement of high quality in health services and addressed in professional policies [57]. One of the most important gains of working in a team is developing the person’s own competence in relation to other professionals’ competence. Competence was closely linked to the PTs’ ability to reflect on the profession, professional practice and individual competence, something that was found in all the themes. This ability seems applicable in the development of all competences [58] and was expressed as a central aspect of developing competence. Edwards [59] focuses on the relational aspects of competence, meaning that the individual competence of a practitioner is inherently bound to the competence of other practitioners. Indeed, competence is a collective activity, and the goal within a workplace should be for the staff to reciprocally strengthen each other’s competences so that the amount of collective competence is larger than the sum of individual competences. Here, competence includes a mixture of knowledge, skills and personal abilities, as well as the relational and contextual aspects of competence in community care of the elderly. Therefore, having a well-educated staff that can competently meet the needs of elderly patients is essential [60]. Our informants emphasised that using experiences in a knowledgeable and reflective manner seemed like an especially fruitful strategy for enhancing the development of competence. The results clearly displayed the importance of competences, such as communicational, behavioural and reflective skills, and appeared to place these skills above the traditionally highly valued medical or technical skills endorsed by today’s educational system [61]. One key aspect of how competence was used in practice is connected to experience-based knowledge among the PTs and the statement that the *PTs’are concerned about improving their level of scientific understanding and want to perform evidence –based practice* might indicate that. Similarities can be found in other research in this area [62, 63], pointing to the need for PTs to incorporate communicational and cooperative competences to utilise their competences adequately. There are several other important factors, such as personal confidence, that can strongly influence the PTs’ competence, that the current study does not elucidate. However, their impact on competence should not be disregarded. In addition, the perception of competence is closely linked to patient outcomes [15, 16]. This link between perceptions of competence and patient outcomes could be explained by the general emphasis on the problem-solving competence by the professionals themselves, as shown in earlier research [57].

Finally, our informants were concerned about structural constraints and frustrated over the poor economic framework that they had to work within. They noted that there were too few employees with poor resources, especially in some localities. The PTs wanted to have an impact on the decisions made by *politicians and those who are responsible for* the allocation of financial resources. These results are in line with those by Lipsky [64], who describes how frontline providers, or ‘street-level bureaucrats’, often work with conflicting and ambiguous goals because their tasks might be characterised by the tension among the patients’ needs, available resources and regulatory constraints, that is, the conflicting institutional logics. The healthcare professionals who meet the patients face to face are actually those who perform the policy that is first decided on at a higher level, and these practitioners are the ones who try to find acceptable strategies to deal with this policy in their practice.

### Strengths and limitations

The present study is built upon a large database, with ‘thick’ descriptions of the therapeutic alliance as a phenomenon. We analysed a small part of the original amount of the data, and we may have missed out important elements in the data analysis. Furthermore, we recognise that including a larger number of participants who represent different geographical regions can yield a wider range of perspectives on the important determinants that influence a therapeutic alliance. Furthermore, the findings from the urban institutions might not be transferable to health services in rural districts or in other countries. In addition, particular groups of therapists, such as therapists from ethnic minorities, were not included in the sample.

## Conclusion

Our findings indicate that the PTs’ role reflects their abilities to change and improve their professional work in accordance with evidence based knowledge. To ensure good quality the PTs focused on the special needs of the patients, evidence-based fall prevention, interdisciplinary team work, good clinical competences, good skills in communication, and interpersonal relations. Exploring the view of patients or other members of the interdisciplinary fall prevention team on their role towards fall prevention in the community will be of great interest in the future.

## Data Availability

The datasets generated and analysed during the current study are not publicly available due to protect participant confidentiality but are available from the corresponding author on reasonable request.

## Abbreviations

EBP: Evidence-based practice
GP: General practitioners
OEP: Otago exercise programme
PT: Physiotherapists
